# Generalisierbarkeit von Phase III Klinischen Studien am Beispiel Zweier Bundesweiter Multiple Sklerose Register

**DOI:** 10.1055/a-2540-1749

**Published:** 2025-07-23

**Authors:** Kris Oliver Jalusic, David Ellenberger, Alexander Stahmann, Klaus Berger

**Affiliations:** 1 Institut für Epidemiologie und Sozialmedizin, Universität Münster Medizinische Fakultät Münster, Münster, Germany; 2 Deutsches MS Register, MS Forschungs- und Projektentwicklungs-gGmbH, Hannover, Germany; 3 Institut für Epidemiologie und Sozialmedizin, Universität Münster, Münster, Germany

**Keywords:** Epidemiologie, Pharmakoepidemiologie, Multiple Sklerose, Epidemiology, Multiple Sclerosis, Pharmacoepidemiology

## Abstract

**Hintergrund:**

Neu zugelassene Therapien haben meist noch unbekannte Nebenwirkungen, obwohl die klinischen Studien, die zur Zulassung führten, bereits Sicherheit und Wirksamkeit analysierten. Ein Grund dafür ist, dass die Ein- und Ausschlusskriterien der Studien das meist heterogenere Patientenkollektiv in der klinischen Routineversorgung oft nicht komplett abbilden.

**Fragestellung:**

In dieser Studie wurden die Auswirkungen der Übertragbarkeit von Phase-III Ein- und Ausschlusskriterien auf MS-Patienten in der klinischen Praxis analysiert, die mit DMDs behandelt werden. Dabei wurden die demografischen und klinischen Merkmale bei Therapiebeginn zwischen Patienten verglichen, die alle Kriterien erfüllt hätten und solchen, die mindestens eines nicht erfüllt hätten. Zudem wurden Unterschiede in der Häufigkeit von (schwerwiegenden) unerwünschten Ereignissen ((S)UEs) zwischen den beiden Gruppen untersucht.

**Methoden:**

Datenbasis bildeten zwei nationale, prospektive, beobachtende, klinische, multizentrische Register, das REGIMS-Register und das MS-Register der DMSG. Folgende Ein- bzw. Ausschlusskriterien wurden angewandt: Alter, klinischer Verlauf der RRMS, Schübe, EDSS-Score, Medikationsgeschichte. Für kategoriale Vergleiche wurde der Chi-Quadrat-Test durchgeführt und für kontinuierliche Variablen der t-Test. Um den Unterschied in den Daten zur (S)UEs zu untersuchen, wurden logistische Regressionsmodelle berechnet. Ein p-Wert von<0,05 wurde als statistisch signifikant angesehen.

**Ergebnisse:**

28% der Patienten des REGIMS-Registers und 5% der Patienten des MS-Registers haben die 4 vordefinierten Einschlusskriterien erfüllt und wären somit in eine Phase-III-Zulassungsstudie der entsprechenden Substanz aufgenommen worden.

**Schlussfolgerung:**

Unsere Ergebnisse zeigen eine deutliche Patientenselektion durch spezifische Einschlusskriterien in klinischen Studien von MS-Therapeutika, verglichen mit dem Patientenkollektiv, das nach Zulassung diese Therapie erhält. Diese Selektion geht allerdings nicht mit einem höheren Risiko in Bezug auf SUEs für diejenigen Patienten einher, die nicht in die entsprechende klinische Phase-III-Studie eingeschlossen worden wären.

## Abstract (en)

**Background:**

Newly approved therapies usually have unknown adverse events, although the clinical trials that led to approval had already tested them for safety and efficacy. One reason for this is that the inclusion and exclusion criteria of the trials often do not fully reflect the usually heterogeneous patient population in routine clinical care.

**Aim:**

The aim of the study was to analyse the extent to which patients with multiple sclerosis (MS) in routine clinical care fulfil the inclusion and exclusion criteria for the corresponding clinical phase III trial of the respective drug.

**Methods:**

Sociodemographic and clinical characteristics as well as (serious) adverse events ((S) AEs) were compared. Data were based on two national, prospective, observational, clinical, multicentre registries, the REGIMS registry and the DMSG MS registry.

**Results:**

Patients (28%) in the REGIMS registry and 5% of the patients in the MS registry fulfilled the 4 predefined inclusion criteria and would therefore have been included in a phase III registration trial of the corresponding substance.

**Conclusion:**

Our results show a clear selection of patients by specific inclusion criteria in clinical trials of MS therapeutics compared to the patient population receiving this therapy after approval. However, this selection is not associated with a higher risk of AEs for those patients who would not have been included in the corresponding phase III clinical trial.

## Einleitung

Multiple Sklerose ist eine chronische Autoimmunerkrankung des zentralen Nervensystems. Die klinischen Symptome sind heterogen, aber bei den meisten Patienten ist das Anfangsstadium durch (teil)reversible Episoden neurologischer Funktionsstörungen gekennzeichnet, gefolgt von irreversiblen klinischen und kognitiven Defiziten 1 .

Die Mehrzahl der MS-Patienten werden mit krankheitsmodifizierenden Medikamenten (DMD) behandelt, um Schübe und ein Fortschreiten der Krankheit zu verhindern 2 . Der Markt für MS-Medikamente zur Behandlung der aktiven MS hat sich rasant entwickelt. In den letzten 10 Jahren wurden fast jährlich neue Medikamente zugelassen, so dass die Behandlungsmöglichkeiten stetig zunehmen 3 . MS ist daher ein Beispiel für einen dynamischen Markt, der mit bekannten Herausforderungen im Bereich der Pharmakovigilanz konfrontiert ist. Zu letzteren gehört die Frage der Generalisierbarkeit der Ergebnisse der Phase-III-Zulassungsstudien auf alle später behandelten Patienten mit der Krankheit, die seit langem Gegenstand kontroverser Diskussionen zwischen klinischen Prüfern und den Behörden ist, die neue Medikamente zulassen. Die Generalisierbarkeit umfasst u. a. den Aspekt der Wirksamkeit des neu zugelassenen Arzneimittels bei Patienten, die die Einschlusskriterien der entsprechenden Zulassungsstudie nicht erfüllen. Sie werden z. B. aufgrund ihres Alters, ihrer Begleiterkrankungen oder spezifischer Krankheitsmerkmale von Phase-III-Studien ausgeschlossen, aber in der Regelversorgung, dem klinischen Alltag, mit dem zugelassenen Arzneimittel behandelt 4 . Hinter der Frage der Generalisierbarkeit steht so auch die mittel- und langfristige Sicherheit eines neuen Medikaments, insbesondere in dynamischen Märkten, in denen die Behandlungserfahrung mit vielen der neu zugelassenen Medikamente noch begrenzt ist.

Phase-III-Studien liefern Informationen über die Wirksamkeit und Sicherheit für viele häufige, unerwünschte Ereignisse (UEs) und sind daher für die Arzneimittelzulassung unerlässlich 5 . Allerdings können in Phase-III-Studien, seltene (schwerewiegende) (S)UEs nicht zuverlässig erkannt werden. Darüber hinaus kann die in klinischen Phase-III-Studien mit strengen Ein- und Ausschlusskriterien analysierte Arzneimittelsicherheit das Sicherheitsprofil in der breiten klinischen Anwendung nach der Arzneimittelzulassung nur unzureichend abbilden 6 . Aufgrund existierender Ein- und Ausschlusskriterien in klinischen Studien der Phase III kann die Übertragung der Ergebnisse in die klinische Routine eingeschränkt sein 7 .

Ziel unserer Untersuchung war es, zu analysieren, wie viele MS-Patienten, die in der klinischen Routineversorgung mit einem DMD behandelt werden, die Ein- und Ausschlusskriterien der zur Zulassung führenden klinischen Phase-III-Studie erfüllt hätten und somit potenzielle Studienteilnehmende gewesen wären. Ferner haben wir Patienten, die Ein- und Ausschlusskriterien erfüllen, mit denen verglichen, die diese Kriterien nicht erfüllen und Unterschiede in soziodemographischen und klinischen Merkmalen sowie (S)UEs analysiert.

## Methoden

Für diese Studie wurden Daten des REGIMS-Registers 8 und des MS-Registers der Deutschen Multiple Sklerose Gesellschaft 9 ausgewertet. Es wurden MS-Patienten mit einer Behandlung durch DMDs die bis 2018 zugelassen worden waren, eingeschlossen: Ocrelizumab, Cladribin, Daclizumab, Dimethylfumarat, Teriflunomid, Alemtuzumab, Fingolimod, Natalizumab, Mitoxantron, Glatirameracetat, Peginterferon β-1a, Interferon β-1b.

### Ethik

Patienten gaben in beiden Registern eine schriftliche Einverständniserklärung ab. Das REGIMS-Register wurde von der Ethikkommission der Medizinischen Fakultät der Universität Bochum bewilligt (Bewilligungsnummer: 4588-13). Das MS-Register der DMSG wurde von der Ethikkommission der Julius-Maximilians-Universität Würzburg initial bewilligt (Bewilligungsnummer: 142/12).

### Patienten

#### REGIMS

REGIMS war ein Immuntherapie-Register, dass innerhalb des krankheitsbezogenen Kompetenznetzes Multiple Sklerose (KKNMS) vom Bundesministerium für Bildung und Forschung (BMBF) gefördert wurde. REGIMS erfasste die Häufigkeit, Art, Charakteristika und Auswirkungen von (S)UEs bei aktuellen und neuen immuntherapeutischen DMDs, die in der klinischen Routineversorgung von MS-Patienten eingesetzt werden. REGIMS war ein nationales, prospektives, beobachtendes (d. h. nicht-interventionelles), klinisches, multizentrisches Register. Weitere Einzelheiten zu REGIMS wurde an anderer Stelle beschrieben 8 .

In diese Analyse wurden Patienten eingeschlossen, die zwischen Oktober 2013 und September 2021 in REGIMS aufgenommen wurden und eine Behandlung mit einen der oben genannten DMDs begonnen haben. In das REGIMS-Register wurden keine Patienten aufgenommen die mit Interferonen oder Glatirameracetat länger als 36 Monate behandelt wurden. Aus den 1865 REGIMS-Patienten, die eine DMD-Behandlung erhielten, wurden aufgrund der geringen Anzahl von Patienten mit PPMS und SPMS ausgeschlossen. Weiterhin wurden Patienten mit unvollständigen Daten über den Beginn der DMD-Behandlung, fehlendem EDSS-Wert (Expanded Disability Status Scale), Daten über Schübe und unplausible Daten über die Medikationsgeschichte zusätzlich ausgeschlossen, so dass in die Auswertung 1271 Patienten einbezogen wurden. Davon hatten 1252 mindestens eine Nachuntersuchung mit Daten zur Arzneimittelsicherheit.

#### MS-Register

Das MS-Register wurde 2001 von der Deutschen Multiple Sklerose Gesellschaft, Bundesverband e.V. (DMSG) initiiert, um bundesweit, einheitlich Daten von MS-Patienten und deren Versorgungssituation zu erheben 10 . Das MS-Register baut auf einem bundesweiten Netzwerk von DMSG-ausgezeichneten MS-Zentren auf und erfasst gesundheitsbezogene Informationen hinsichtlich Demographie, Krankheitsaktivität und -progression, medikamentöse und nicht-medikamentöse Therapien, sowie Pflege der MS-Patienten. Im Jahr 2019 wurde in Kooperation mit dem Institut für Epidemiologie und Sozialmedizin der Universität Münster (Koordinatorin des REGIMS-Registers) ein Modul zur Dokumentation der Pharmakovigilanz innerhalb des MS-Registers gestartet. Abgrenzend zu REGIMS wurden die weiten Einschlusskriterien des MS-Registers auch für die PV-Kohorte innerhalb des MS-Registers beibehalten, so dass auch Patienten auf langjährigen Basistherapien, in Therapiepausen oder vor Therapiebeginn (naive) eingeschlossen werden können. Patienten, die bereits zu dem Zeitpunkt des PV-Beobachtungsstarts eine Therapie bekommen, werden mit dem ersten PV-Follow-Up in die PV-Kohorte eingeschlossen. Weitere Einzelheiten zum MS-Register der DMSG wurden an anderer Stelle beschrieben 9 .

Das MS-Register umfasst zum Zeitpunkt der Analyse (Datenstand 4.4.2023) seit seiner Initiierung im Jahr 2001 Daten von über 80 000 Patienten. Für den Einschluss in die Analyse musste der EDSS-Wert, der klinische Verlauf, Daten über Schübe und über die vorherige Medikationsgeschichte verfügbar sein. Da das Pharmakovigilanz-Modul (N _PV_ =4579) nachträglich in das MS-Register integriert wurde, schloss diese Analyse nur Patienten ein, die seit dem Beginn der Teilnahme des jeweiligen behandelnden Zentrums am Modul therapiert wurden, wodurch insgesamt N=1191 MS-Register-Patienten einbezogen wurden.

### Datenanalyse

Die Ein- und Ausschlusskriterien wurden aus den klinischen Phase-III-Studien und den von der Europäischen Arzneimittelagentur veröffentlichten Zusammenfassung der Produktmerkmale für jedes DMD extrahiert. In den meisten Phase-III-Studien wurden Patienten eingeschlossen, die zwischen 18 und 55 Jahre alt waren (Kriterium Alter), einen klinischen Verlauf der RRMS mit mindestens zwei Schüben innerhalb der letzten zwei Jahre vor der Randomisierung oder einem Schub im Jahr davor hatten (Kriterium Schübe) und/oder eine MRT-Untersuchung des Gehirns, die auf MS hindeutet (z. B. mindestens eine Gadolinium-verstärkte Läsion 0 bis 6 Wochen vor der Randomisierung). Da die genaue Anzahl der T2-Läsionen weder im REGIMS- noch im MS-Register erfasst wird, wurde das Kriterium zur Magnetresonanztomographie (MRT) nicht in Betracht gezogen. Ein weiteres Auswahlkriterium war ein EDSS-Score zwischen 0.0 und 5.0 (Kriterium EDSS-Score). Das **Onlinematerial 1** zeigt eine Zusammenfassung der angewandten Einschlusskriterien. Da in dieser Arbeit nur RRMS Patienten analysiert wurden, wurde das Kriterium MS-Verlaufsform nicht angewandt. Die Medikationsgeschichte ist ein häufiges Ausschlusskriterium in klinischen Studien ( **Onlinematerial 2** ).

In einem ersten Schritt wurde der Anteil an Patienten analysiert, die in eine klinische Phase-III-Studie hätten eingeschlossen werden können. Im nächsten Schritt wurde der Prozentsatz der Patienten, die jedes Kriterium der entsprechenden Studie erfüllten je DMD separat, berechnet. Schließlich wurden die Patienten, die alle Ein- und Ausschlusskriterien für ein bestimmtes Medikament erfüllten, mit denen, die diese Kriterien nicht erfüllten, hinsichtlich klinischer und soziodemografischer Merkmale verglichen, in diesen Vergleich wurden folgende Variablen einbezogen: Alter bei Therapiebeginn und bei Diagnosestellung, EDSS-Score bei Therapiebeginn und die Anzahl der Schübe 24 Monate vor Therapiebeginn. Zusätzlich wurde die Häufigkeit des Auftretens von (S)UEs unter Therapie verglichen. Die Dokumentation der (S)UEs in den Registern erfolgt(e) in einem elektronischen Datenerfassungssystem (EDC) durch die Registerzentren. (S)UEs wurden in beiden Registern unabhängig von der Kausalität gemeldet. SUEs wurden sofort an die Register weitergeleitet und dem Zulassungsinhaber gemeldet. Schübe werden nicht als UEs dokumentiert, sondern als Krankheitsprogression bewertet. Analysiert wurden (S)UEs welche nach Therapiebeginn neu auftraten über einen Nachbeobachtungszeitraum von bis zu 30 Monaten nach Therapiebeginn für die REGIMS-Patienten und 51 Monate für MS-Register-Patienten.

Die Patienten beider Register wurden anhand der MS spezifischen Therapie in 3 Gruppen unterteilt. Die Einteilung basiert auf der Sk2 Leitlinie der Deutschen Gesellschaft für Neurologie (DGN) 11 . In dieser sind DMDs in 3 Wirksamkeitskategorien in Abhängigkeit von der relativen Reduktion der entzündlichen Aktivität unterteilt. Dabei ist die stärkste Reduktion der entzündlichen Aktivität in der Kategorie 3 zu beobachten. Zu beachten ist, dass es keine kontrollierten Head-to-Head-Vergleichsstudien zwischen den unterteilten DMDs gibt und die Kategorien nicht als Therapiesequenzen zu interpretieren sind. In dieser Analyse wurde die Einteilung der betrachteten DMDs wie folgt durchgeführt: Wirksamkeitskategorie 1 (Dimethylfumarat, Glatirameracetat, Interferon-beta, Teriflunomid); Wirksamkeitskategorie 2 (Cladribin, Fingolimod); Wirksamkeitskategorie 3 (Alemtuzumab, Natalizumab, Ocrelizumab); ohne Kategorie (Mitoxantron). Daclizumab wird in der Sk2 Leitlinie nicht länger in Betracht gezogen und wurde in dieser Arbeit der Kategorie 3 zugefügt.

Für kategoriale Vergleiche zwischen den Gruppen der Patienten, die in eine Studie eingeschlossen wären und die mindestens ein Kriterium nicht erfüllt haben, wurde der Chi-Quadrat-Test durchgeführt und für kontinuierliche Variablen der t-Test. Um den Unterschied in den Daten zur (S)UEs zwischen Patienten, die alle vier vordefinierten Ein- und Ausschlusskriterien erfüllten, und solchen, die diese nicht erfüllt haben, zu untersuchen, wurden logistische Regressionsmodelle berechnet. Ein p-Wert von<0,05 wurde als statistisch signifikant angesehen.

## Ergebnisse

### Demographische und klinische Charakteristika

In den beiden Registern ist eine vergleichbare Geschlechterverteilung zu beobachten. 71% der 1271 REGIMS-Patienten und 72% der 1191 Patienten des MS Registers sind weiblich. Das durchschnittliche Alter der Patienten zum Beobachtungszeitpunkt ist im MS-Register etwas höher (41 Jahre vs. 37 Jahre). Des Weiteren weisen REGIMS-Patienten im Verglich zu den Patienten des MS-Registers eine höhere durchschnittliche jährliche Schubrate (1,5 vs. 0,3) und einen höheren EDSS-Wert (2,6 vs. 2,5) zum Zeitpunkt der Therapieinitiierung auf. Das Patientenkollektiv des MS-Registers hatte eine mediane Erkrankungsdauer von 13 Jahren, bei REGIMS-Patienten betrug diese 8 Jahre ( Tab. 1 ).

**Table:** **Tab. 1** Demographische und klinische Merkmale der Patienten.

Charakteristika	REGIMS			MS REGISTER (DMSG)		
	Total	Alle Kriterien erfüllt	Nicht alle Kriterien erfüllt	Total	Alle Kriterien erfüllt	Nicht alle Kriterien erfüllt
Patienten (N).	1271	354	917	1191	61	1130
Weiblich, (%)	70,7	68,4	71,7	71,8	73,8	71,7
Alter, Jahre, Mittelwert (SD)	36,9 (10.8)	34,1 (8,9)	38,0 (11,3)	40,9 (11,7)	38,3 (8,9)	41,0 (11,9)
Alter, 1. Symptome Mittelwert (SD)	29,3 (10,0)	28,0 (8,5)	29,8 (10,4)	31,0 (10,3)	28,0 (8,4)	31,2 (10,3)
Diagnosealter, Jahre, Mittelwert (SD)	31,2 (10,3)	29,7 (8,9)	31,8 (10,7)	33,0 (10,5)	29,8 (9,0)	33,1 (10,6)
Erkrankungsdauer, Mittelwert (SD)	7,7 (6,5)	5,7 (5,4)	8,5 (6,7)	12.5 (8,5)	13,1 (9,0)	12,5 (8,5)
EDSS-Score, Mittelwert (SD)	2,6 (1,6)	2,3 (1,2)	2,7 (1,7)	2,5 (1,7)	2,5 (1,3)	2,5 (1,8)
Jährliche Schubrate ^a^ , Mittelwert (SD)	1,5 (1,6)	2,7 (1,6)	1,0 (1,3)	0,3 (0,7)	1,6 (0,9)	0,3 (0,6)

DMD: disease-modifying drug; EDSS: expanded disability status scale; RRMS: relapsing-remitting multiple sclerosis, ^a^ Anzahl der Schübe 24 Monate vor Therapieinitiierung.

**Abb. 1:**
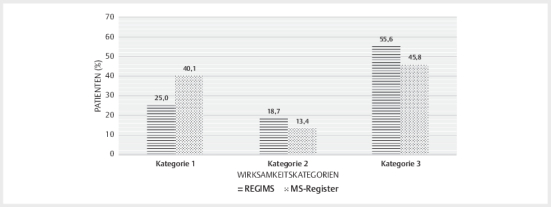
Wirksamkeitskategorien der analysierten Therapien. Wirksamkeitskategorie 1: Dimethylfumarat, Glatirameracetat; Interferone, Teriflunomid. Wirksamkeitskategorie 2: Cladribin, Fingolimod; Wirksamkeitskategorie 3: Alemtuzumab, Natalizumab, Ocrelizumab.

Die Patienten des REGIMS-Registers wurden durchschnittlich mit 1,4 DMDs vor der analysierten Therapie behandelt, darunter waren sowohl de-novo-Patienten als auch Patienten mit bis zu 13 Vortherapien. Patienten des MS-Registers wurden vor der untersuchten Therapie mit durchschnittlich 1,2 DMDs behandelt und im Maximum gab es einen Patienten mit bis zu 10 Vortherapien. Zur untersuchten Behandlungsepisode wurden 55,6% der Patienten im REGIMS-Register und 45,8% der Patienten im MS-Register mit einem DMD der Kategorie III therapiert. Im MS-Register erhielten 40,1% der Patienten ein DMD der Wirksamkeitskategorie I, während 25,0% der Patienten im REGIMS-Register mit einem DMD dieser Kategorie behandelt wurden ( Abb. 1 ).

### Erfüllung der Einschlusskriterien von Phase-III-Studien nach der Zulassung

#### REGIMS

27,9% der 1271 analysierten Patienten des REGIMS-Registers haben die 4 vordefinierten Kriterien erfüllt und wären somit in eine Phase-III-Zulassungsstudie aufgenommen worden ( Tab. 2 ). Dabei wurde das Kriterium Schub, mit 39,4% am seltensten erfüllt, gefolgt vom Kriterium Medikationsgeschichte (74,4%). Umgekehrt hätten die meisten Patienten das Kriterium EDSS und Alter (92,1%) erfüllt. Wäre das Kriterium Schub ausgeschlossen worden, hätten 65,1% der REGIMS-Patienten die drei Kriterien Alter, Medikationsgeschichte und EDSS-Wert erfüllt.

**Table:** **Tab. 2** Prozent der Patienten, die die Einschlusskriterien der entsprechenden Phase-III-Studie erfüllten, nach Substanz.

DMD	Patienten (N)		Einschlusskriterien								Alle Kriterien erfüllt (% der Patienten)		Drei Kriterien erfüllt (% der Patienten) ^a^	
			Alter		EDSS-Score		Schübe		Medikationsgeschichte					
	REG.	DMSG	REGIMS	DMSG	REGIMS	DMSG	REGIMS	DMSG	REGIMS	DMSG	REGIMS	DMSG	REGIMS	DMSG
Alle DMDs	1271	1191	92,1	87,2	92,1	90,4	39,4	12,3	74,4	72,2	27,9	5,1	65,1	60,2
Ocrelizumab	94	375	92,6	89,7	88,3	80,0	36,2	12,5	70,2	54,9	25,5	2,7	60,6	44,8
Cladribin	17	72	100	100	94,1	87,5	47,1	23,6	76,5	45,8	41,2	5,6	76,5	37,5
Daclizumab	9	1	100,0	100,0	88,9	0,0	66,7	0,0	55,6	1,0	33,3	0,0	55,6	0,0
Dimethylfumarat	116	148	92,2	92,6	92,2	99,3	29,3	8,1	56,9	78,4	15,5	4,7	49,1	73,0
Teriflunomid	59	117	78,0	76,2	93,2	97,4	20,3	14,5	69,5	81,2	11,9	7,7	50,9	64,1
Alemtuzumab	166	44	98,8	100	92,2	93,2	63,3	13,6	88,6	88,6	52,4	9,1	82,5	81,8
Fingolimod	220	87	94,6	90,8	95,0	95,4	37,7	3,5	n/a	n/a	34,1	3,5	90,0	87,4
Natalizumab	447	125	89,5	86,4	89,9	92,8	34,0	24,0	57,7	88,0	16,3	16	48,1	73,6
Mitoxantron	n/a	9	n/a	44,4	n/a	66,7	n/a	100,0	n/a	66,7	n/a	22,2	n/a	22,2
Glatirameracetat	71	109	87,3	96,3	95,8	2,8	28,2	2,8	n/a	n/a	26,8	1,8	84,5	72,5
Interferone	72	104	97,2	84,6	97,2	98,1	65,3	1,9	80,6	55,8	56,9	0	76,4	51,9

EDSS Score: expanded disability status scale; ^a^ ohne das Kriterium Schübe

#### MS Register

5% der 1191 Patienten des MS-Registers hätten die 4 vordefinierte Kriterien erfüllt und wären somit in eine Phase-III-Zulassungsstudie aufgenommen worden ( Tab. 2 ). Die meisten Patienten des MS-Registers hätten das Kriterium EDSS Wert erfüllt (90,4%), gefolgt vom Alter (87,2%) und Medikationsgeschichte (72,2%). Die Analyse ohne das Kriterium Schub zeigt, dass 60,2% der Patienten des MS-Registers die restlichen drei definierten Kriterien erfüllt hätten.

### Patientenvergleich nach Erfüllung von Einschlusskriterien

#### Regims

Patienten, die alle Kriterien erfüllt hätten, waren durchschnittlich 3 Jahre älter (SD: 0,7, p<,001), hatten einen um 0,4 Punkte (SD: 0,1, p<,001) höheren EDSS-Wert und 2 Schübe (SD: 0,8, p<,001) vor Therapiebeginn weniger. Tab. 3 zeigt einen Vergleich von klinischen und demographischen Charakteristika von Patienten, die die vier vordefinierten Kriterien erfüllt hätten und der Patienten, die eines oder mehr nicht erfüllt hätten für die häufigsten Medikamente im REGIMS-Register. Die Analyse für die jeweiligen Medikamente (mit Ausnahme von Cladribin und Dimethylfumarat) zeigt einen ähnlichen Trend wie die therapieübergreifende Analyse. Tab. 3 vergleicht Patienten, die das Kriterium Alter, EDSS-Wert und Medikationsgeschichte erfüllt hätten mit Patienten, die eines der drei Kriterien nicht erfüllt hätten.

**Table:** **Tab. 3** Differenz in Mittelwerten von Patientencharakteristika zwischen denen, die alle Einschlusskriterien für Phase-III-Studien erfüllen im Vergleich zu Patienten, die mindestens ein Kriterium nicht erfüllen, nach Substanz.

Mittelwert Δ	Ocrelizumab	Cladribin	Daclizumab	Dimethyl- fumarat	Teriflunomide	Alemtuzumab	Fingolimod	Natalizumab	Mitoxantron	Glatiramer acetat	Interferone
REGIMS											
Kriterien	Alter, Schübe, EDSS-Wert, Medikationsgeschichte										
Patienten ^a^	70	10	6	98	52	79	145	374	n/a	53	31
Alter (Jahre)	+5,9*	–5,1	+6,8	+4,0	+7,7	+2,4	+4,0**	+4,2**	n/a	+6,2*	+3,8
Alter bei Diagnose (Jahre)	+3,0	–8,7	+3,7	+1,5	+5,0	+0,2	+2,6	+3,0*	n/a	+6,6*	–0,9
EDSS-Wert	+0,6	+0,1	+2,3	–0,1	+0,8	+0,5*	+0,1	+0,5*	n/a	+0,4	+0,8*
Schübe 24 Monate, Anzahl	–1,8***	–1,7**	–1,5*	–1,8***	–1,3**	–2,0***	–2,1***	–1,6***	n/a	–1,6***	–0,4
Kriterien	Alter, EDSS-Wert, Medikationsgeschichte										
Patienten ^a^	37	4	4	59	29	79	22	232	n/a	11	17
Alter (Jahre)	+6,7**	–6,9	+19,3**	+5,0*	+10,4***	+2,4	+13,7***	+4,4***	n/a	+20,3***	+4,1
Alter bei Diagnose (Jahre)	–0,4	–10,3	+12,0	+1,7	+7,7*	+0,2	+7,8***	+3,6***	n/a	+21,4***	-2,0
EDSS-Wert	+1,6***	+7,5	+3,2**	+0,8*	+0,9*	+0,5*	+2,5***	+0,9***	n/a	+1,6****	+1,3***
Schübe 24 Monate, Anzahl	–0,5	–0,3	–0,2	–0,4	0,0	–2,0***	0,0	0,0	n/a	–0,6*	+0,2
MS REGISTER (DMSG)											
Kriterien	Alter, EDSS-Wert, Medikationsgeschichte										
Patienten ^a^	207	45	n/a	40	42	36	76	92	n/a	79	54
Alter (Jahre)	+8,8***	+1,5	n/a	+6,8***	+12,1***	+0,3	+14,8***	+15,2***	n/a	+21,4***	+10,8***
Alter bei Diagnose (Jahre)	+2,7***	–2,9	n/a	+3,4*	+6,4**	0	+11,0**	+11,6***	n/a	+16,4***	+5,5*
EDSS-Wert	+2,5*	+0,9*	n/a	+2,8	+1,1***	+2,4***	+2,2***	+1,7***	n/a	+1,1***	+0,6*
Schübe 24 Monate, Antzahl	+0,3***	0,1	n/a	+0,1	+0,1	+0,5	–0,3	+0,2	n/a	0	+0,1

^a^ Zahl der Patienten die mindestens ein Kriterium nicht erfüllt haben; DMD: disease-modifying drug; EDSS: expanded disability status scale; * *p* <0,05; ** *p* <0,01; *** *p* <0,001

#### MS-Register

Patienten des MS-Registers die alle Kriterien erfüllt hätten waren durchschnittlich 2.6 Jahre älter (SD: 1,5, p=0,09), hatten einen vergleichbaren EDSS-Wert und einen Schub weniger (SD: 0,08, p<,001) vor Therapiebeginn. Wegen der wenigen Patienten, die die vier vordefinierten Kriterien erfüllt hätten, wurde kein DMD-spezifischer Vergleich mit Patienten, die ein oder mehr Kriterien nicht erfüllten berechnet. Tab. 3 vergleicht Patienten, die die Kriterien Alter, EDSS-Wert und Medikationsgeschichte erfüllt hätten mit Patienten, die eines der drei Kriterien nicht erfüllt hätten. Dabei ist zu beobachten, dass die Patienten, die mindestens eines der drei Kriterien erfüllt hätten, älter sind, einen höheren EDSS-Wert haben und eine höhere Schubaktivität aufweisen.

### Vergleich von (S)UEs

#### Regims

41,5% der REGIMS-Register Patienten hatten mindestens ein UE und 8,5% ein SUE im Beobachtungszeitraum von bis zu 30 Monaten. Patienten/-innen, die die vier vordefinierten Kriterien erfüllt hätten, berichteten mehr UEs (47,1% vs. 39,3%) und SUEs (9,4% vs. 8,1%) als diejenigen, die nicht alle Kriterien erfüllten. Bei der Betrachtung der UEs lässt sich dieser Trend auch für Ocrelizumab, Alemtuzumab, Fingolimod und Natalizumab erkennen. Umgekehrt hatten Patienten, die mit Dimethylfumarat behandelt wurden und nicht alle vier Kriterien erfüllt hätten, mehr UEs (29,9% vs. 23,5%), während mit Ocrelizumab (2,9% vs. 0,0%) und Alemtuzumab (14,5% vs. 11,9%) therapierte Patienten/-innen, mehr SUEs Angaben ( Tab. 4 ). In einer weiteren Analyse, bei der Schübe nicht als Einschlusskriterium berücksichtigt wurden, war der Anteil der Patienten mit mindestens einem UE (42,1% vs. 40,3%) bei der Patientengruppe, die alle Kriterien erfüllt hätte, etwas höher. Bei der Betrachtung der SUEs ist dieser Trend umgekehrt: bei Patienten, die nicht alle der Kriterien erfüllt hätten konnte ein höherer Patientenanteil mit SUEs (9,4% vs 8%) beobachtet werden ( Tab. 4 ).

**Table:** **Tab. 4** Vergleich der Häufigkeit der (schwerwiegenden) unerwünschten Ereignisse.

Disease modifying drug (DMD)	All DMDs		Ocrelizumab		Dimethylfumarat		Alemtuzumab		Fingolimod		Natalizumab	
Kriterien	Alter, Schübe, EDSS-Wert, Medikationsgeschichte											
4 Kriterien sind erfüllt	Ja	Nein	Ja	Nein	Ja	Nein	Ja	Nein	Ja	Nein	Ja	Nein
REGIMS (Patienten [N])	350	902	24	69	18	97	85	76	74	143	72	373
Unerwünschtes Ereignis (UE) [%]	47,1	39,3	25,0	17,4	22,2	30,5	62,4	59,2	59,5	49,0	40,3	37,0
Schwerewiegendes unerwünschtes Ereignis (SUE) [%]	9,4	8,1	0	2,9	11,1	6,3	11,8	14,5	16,2	11,9	8,3	7,5
MS-Register (Patienten [N])	61	1130	10	365	7	141	4	40	3	84	20	105
Unerwünschtes Ereignis (UE) [%]	18,0	13,1	20,0	11,7	0,0	14,9	0,0	7,5	33,3	15,5	30,0	22,9
Schwerewiegendes unerwünschtes Ereignis (SUE) [%]	1,6	1,9	0,0	2,5	0,0	1,4	0,0	7,5	0,0	3,6	5,0	1,9
Kriterien	Alter, EDSS-Wert, Medikationsgeschichte											
3 Kriterien sind erfüllt	Ja	Nein	Ja	Nein	Ja	Nein	Ja	Nein	Ja	Nein	Ja	Nein
REGIMS (Patienten [N])	815	437	57	49	56	57	134	33	195	70	213	232
Unerwünschtes Ereignis (UE) [%]	42,1	40,3	15,8	25,0	26,8	31,6	58,2	74,1	53,9	40,9	33,3	41,4
Schwerewiegendes unerwünschtes Ereignis (SUE) [%]	8,0	9,4	0,0	5,6	5,4	8,8	11,9	18,5	12,3	12,8	6,6	8,6
MS-Register (Patienten [N])	717	474	168	207	108	40	36	8	76	11	92	33
Unerwünschtes Ereignis (UE) [%]	15,3	10,1	16,7	8,2	14,8	12,5	8,3	0,0	17,1	9,1	27,2	15,2
Schwerewiegendes unerwünschtes Ereignis (SUE) [%]	1,7	2,3	1,2	3,4	1,9	0,0	2,8	25,0	4,0	0,0	3,3	0,0

#### MS-Register

Im Pharmakovigilanzmodul des MS-Registers hatten 13.3% der eingeschlossenen Patienten mindestens ein UE und 1,9% ein SUE innerhalb des Nachbeobachtungszeitraums von bis zu 51 Monaten. Medikamentenübergreifend betrachtet war der Anteil der Patienten/-innen, die mindestens ein UE (18,0% vs. 13,0%) berichteten in der Gruppe der Patienten, die alle vier Kriterien erfüllt hätten, höher ( Tab. 4 ) während der Trend bei SUEs (1,6% vs. 1,9%) umgekehrt war. Das konnte auch bei Ocrelizumab-Patienten beobachtet werden. Der Anteil der Dimethylfumarat-(14,9% vs. 0,0%) und Alemtuzumab-(7,5% vs. 0,0%) Patienten mit mindestens einer UE, war in der Gruppe der Patienten, die mindestens ein Kriterium nicht erfüllt hätten, höher. Dieser Trend konnte auch bei der Betrachtung der SUEs für die häufigsten DMDs betrachtet werden, jedoch mit der Ausnahme von Natalizumab-Patienten. Tab. 4 zeigt den Vergleich von UEs und SUEs von Patienten, die drei Kriterien erfüllt hätten (ohne das Kriterium Schub) und die mindestens ein Kriterium nicht erfüllt hätten. Die erste Gruppe hatte einen höheren Anteil der Patienten mit mindestens einem UE (15,3% vs. 10,1%) und einen niedrigeren Anteil der Patienten mit mindestens einem SUE (1,7% vs. 2,3%).

### Regressionsmodelle zur Arzneimittelsicherheit

Tab. 5 zeigt die Ergebnisse der logistischen Regressionsanalyse für das REGIMS-Register und für das MS-Register der DMSG.

**Table:** **Tab. 5** Odds-Ratios für das Risiko eines (schwerwiegenden) eines unerwünschten Ereignisses ((S)UE) basierend auf den einzelnen Einschlusskriterien. Abhängige Variable ist das (S)UE (1=mindestens ein (S)UE; 0=kein (S)UE). Univariat adjustiert auf die Einbeziehung eines einzigen Kriteriums als unabhängige Variable. Multivariat adjustiert auf die gleichzeitige Berücksichtigung aller anderen aufgeführten Kriterien.

UE	Univariat			Multivariat		
REGIMS						
Einschlusskriterien	OR	95% CI	p- Wert	OR	95% CI	p- Wert
Alter	0,818	0,54 0–1,23	0,34	0,81	0,53–1,23	0,32
EDSS-Wert	0,854	0,57–1,29	0,45	0,86	0,56–1,31	0,48
Medikationsgeschichte	1,134	0,88–1,47	0,34	1,11	0,86–1,45	0,43
Schübe	1,523	1,21–1,92	<0,01	1,50	1,21–1,92	<0,01
SUE	Univariat			Multivariat		
Einschlusskriterien	OR	95% CI	p- Wert	OR	95% CI	p- Wert
Alter	0,59	0,32–1,09	0,09	0,64	0,34–1,22	0,18
EDSS-Wert	0,53	0,29–0,98	0,04	0,57	0,30–1,06	0,07
Medikationsgeschichte	1,12	0,70–1,79	0,64	1,56	0,72–1,86	0,55
Schübe	1,25	0,84–1,87	0,28	1,26	0,84–1,89	0,26
MS REGISTER (DMSG)						
UE	Univariat			Multivariat		
Einschlusskriterien	OR	95% CI	p-Wert	OR	95% CI	p-Wert
Alter	2,38	1,22–4,62	0,01	2,03	1,04–3,96	0,04
EDSS-Score	9,49	2,32–38,79	<0,01	8,54	2,07–35,23	<0,01
Medikationsgeschichte	1,16	0,79–1,70	0,45	0,87	0,70–1,53	0,86
Schübe	0,91	0,54–1,54	0,72	1,08	0,63–1,84	0,79
SUE	Univariat			Multivariat		
Einschlusskriterien	OR	95% CI	p-Wert	OR	95% CI	p-Wert
Alter a	n/a	n/a	n/a	n/a	n/a	n/a
EDSS-Score	0,70	0,20–2,39	0,57	0,62	0,17–2,23	0,47
Medikationsgeschichte	0,59	0,25–1,38	0,23	0,61	0,26–1,47	0,27
Schübe	1,08	0,32–3,66	0,90	0,98	0,28–3,43	0,98

^a^ SUE wurden nur bei Patienten beobachtet, die das Einschlusskriterium Alter erfüllen (daher in den Regressionen exkludiert)

Die Ergebnisse der multiplen binären logistischen Regression zeigen, dass Patienten des REGIMS-Registers, die das Kriterium Schübe erfüllt hätten, eine höhere Wahrscheinlichkeit hatten, dass UE auftreten, als diejenigen, die dieses Kriterium nicht erfüllten (OR: 1,5; 95% KI: 1,2–1,9; p<0,001).

Die Analyse zeigte eine statistisch signifikante Assoziation zwischen den UEs und den Kriterien Alter und EDSS-Score bei Patienten des MS-Registers der DMSG. Patienten die das Kriterium Alter (OR: 2,0; 95% KI: 1,0–4,0; p=0,039) und das Kriterium EDSS-Score (OR: 8,5; 95% KI: 2,0–35,2; p<0,003) nicht erfüllt hätten eine höhere Wahrscheinlichkeit hatten, dass UEs auftreten.

## Diskussion

In dieser Studie wurden die Auswirkungen der Übertragung von Ein- und Ausschlusskriterien aus klinischen Studien der Phase III auf MS-Patienten, die in der klinischen Regelversorgung mit DMDs behandelt werden, analysiert. Dafür wurden die demographischen und klinischen Merkmale bei Therapiebeginn zwischen Patienten, die alle Kriterien erfüllt hätten und denen, die mindestens ein Kriterium nicht erfüllt hätten, verglichen. Ferner wurden Unterschiede in der Häufigkeit von (S)UEs zwischen den zwei Gruppen untersucht.

Die Mehrheit der untersuchten MS-Patienten aus der klinischen Routine hätten nicht alle vordefinierten Einschlusskriterien der jeweiligen klinischen Phase-III-Studie erfüllt. 72% der REGIMS-Register Patienten und 95% der betrachteten Patienten im MS-Register der DMSG wären nicht in eine Phase-III-Studie aufgenommen worden. Diese niedrige Übereinstimmung zwischen den Studienpopulationen und Patienten im klinischen Alltag ist ein Ausdruck dafür, das potenzielle Patienten von klinischen Phase-III-Studien, die zur Zulassung von Medikamenten führen, einer strengen Selektion unterliegen.

Schub war das Kriterium, das am seltensten erfüllt wurde. Ohne das Kriterium Schub, steigt der Anteil der Patienten, die alle anderen Kriterien erfüllen, auf durchschnittlich 65% im REGIMS- und 60% im MS-Register der DMSG. Diese Ergebnisse stimmen mit ähnlichen Studien überein, die sich mit der Generalisierbarkeit klinischer Studien in anderen Indikationsfeldern befassen 12 .

Die oben genannten Erfüllungsraten von Phase-III-Zulassungsstudien unterscheiden sich stark zwischen den beiden Registern. Patienten des REGIMS-Registers zeigten eine fast 20% höhere Erfüllungsrate als die Patienten im MS-Register der DMSG. Vor allem das Kriterium Schub würde von den meisten Patienten des MS-Registers der DMSG nicht erfüllt (12,3%). Der Unterschied in der Erfüllung der Kriterien weist auf unterschiedliche demographische und klinische Charakteristika der Patienten/-innen beider Register hin. Die REGIMS-Register-Patienten waren jünger, hatten einen höheren EDSS-Wert und zusätzlich deutlich mehr Schübe, was auf eine höhere Krankheitsaktivität hindeutet. Entsprechend zeigt sich ein höherer Anteil derjenigen, die mit einem hochwirksamen DMD der Kategorie 3 behandelt wurde. Dabei ist zu berücksichtigen, dass in das REGIMS-Register keine Patienten aufgenommen wurden die mit Interferonen oder Glatirameracetat länger als 36 Monate behandelt wurden. Ferner ist zu beachten, dass das Pharmakovigilanz-Modul im MS-Register der DMSG später eingeführt wurde, während die Dokumentation dieser Daten im REGIMS-Register von Beginn durchgeführt wurde. Die aufgeführten Punkte könnte auch die geringere Anzahl von (S)UEs im MS-Register erklären.

Unsere Analyse zeigte, dass die Wahrscheinlichkeit eines UE bei Patienten des REGIMS-Registers, die das Kriterium Schub erfüllen, höher ist als bei Patienten, die dieses Kriterium nicht erfüllen. Die Verringerung der jährlichen Schubrate ist ein häufiger Endpunkt in klinischen Studien und dient als Indikator für die Krankheitsaktivität. Klinische Studien der Phase III schließen Patienten ein, die mindestens einen Schub innerhalb eines Jahres oder mindestens zwei Schübe innerhalb von zwei Jahren vor der Randomisierung hatten und damit eine deutliche Krankheitsaktivität aufweisen. Das Kriterium Schub war das Kriterium mit dem höchsten Prozentsatz an Nichterfüllungen in beiden Registern. Da eine niedrige Schubrate auf eine geringe Krankheitsaktivität hindeutet, kann die Nichterfüllung dieses Kriteriums auf eine konstant erfolgreiche Behandlung hindeuten und wird nicht als potenzielles Sicherheitsrisiko für die Arzneimitteltherapie angesehen.

Neben dem Kriterium Schub zeigten die Patienten die geringste Übereinstimmung mit dem Kriterium Medikationsgeschichte. Washout-Perioden, wie sie in den meisten klinischen Studien der Phase III gefordert werden, sind in der klinischen Routineversorgung kaum zu erreichen. Wenn Patienten nicht auf die aktuelle Behandlung ansprechen oder mindestens einen Schub erleiden, wird in den Leitlinien empfohlen, auf eine Zweitlinientherapie umzusteigen 13 . Sepulveda et al. zeigten außerdem, dass das Absetzen einer Behandlung mit Fingolimod ohne eine geeignete Folgetherapie oft mit einem Auftreten von Krankheitsaktivität assoziiert ist 14 . Diese Verschlechterung wurde ebenfalls beobachtet, wenn die Behandlung mit Natalizumab ohne eine geeignete Folgetherapie beendet wird 15 . Die meisten Patienten werden im Verlauf ihrer Erkrankung mit mehreren DMDs behandelt, sodass insbesondere bei Therapien der Kategorien 2 und 3 de-novo-Patienten selten sind. Unsere Analyse hat gezeigt, dass das nicht erfüllen des Kriteriums Medikationsgeschichte nicht mit einem höheren Risiko in Bezug auf die Arzneimittelsicherheit assoziiert ist.

Limitationen der Studie: Der Ausschluss von Patienten ohne vollständige Daten hat eine kleinere Stichprobengröße für einige DMDs zur Folge, was ein limitierender Faktor dieser Studie ist. Eine weitere Limitation dieser Studie ist, dass die MRT-Daten innerhalb der Register nicht die nötige Granularität aufweisen, so dass dieses Kriterium in der Analyse nicht aufgenommen werden konnte. Dies spiegelt jedoch den klinischen Alltag wieder, da MRTs in der Versorgungspraxis oft nicht standardisiert sind und nur bedingt mit einer Studiensituation verglichen werden können, bspw. Bilder von unterschiedlichen Radiologen ausgewertet werden und longitudinal unterschiedliche Scanner und Protokolle zum Einsatz kommen 16 . COVID Infektionen, die eher bei jüngeren Patienten auftraten, könnten einen Einfluss auf die Regresssionsanalyse der UEs im MS-Registerkollektiv haben. Eine weitere Limitation unserer Studie ist, dass die Erfassung/Dokumentation von Nebenwirkungen setztvoraus, dass diese den Patienten und den dokumentierenden Arzt bekannt sind und war genommen wurden. Des Weiteren werden Ereignisse ungleichmäßig erfasst, da nicht alle Neurologen gleich detailliert beim Patienten nachfragen. Eine Unterschätzung und fehlende Erfassung von (S)UEs ist in PV-Studien häufig und kann auch in dieser Studie nicht ausgeschlossen und als limitierender Faktor interpretiert werden. Eine weitere Limitation dieser Studie ist, dass nur nach AE und SAE unterschieden wurde, aber keine weitere Feingranulierung von verschiedenen Arten von Ereignissen gemacht wurde (z. B. COVID/Infektionskrankheiten gesondert).

Die überwiegende Mehrheit der Patienten mit Multipler Sklerose (MS), die routinemäßig mit einer DMD behandelt werden, wären nicht in die klinischen Studien aufgenommen worden, die zur Zulassung der jeweiligen Medikamente geführt haben. Diese Studie zeigt, dass Patienten, die die rigorosen Auflagen einer Zulassungsstudie nicht erfüllt hätten, idR. kein höheres Risiko haben eine (S)UE zu erleiden. Nur Patienten in einer Datenquelle (MS-Register) die das Kriterium Alter plus das Kriterium EDSS-Score nicht erfüllt hätten weisen eine höhere Wahrscheinlichkeit, dass UEs auftreten auf. Es könnte sinnvoll sein, dass die Zulassungsbehörden und die Studienprüfer ihre Herangehensweise bei der Gestaltung und im Design von Studien neu definieren, damit Studienpopulationen besser die Routineversorgung widerspiegeln. Allerdings können heterogenere Studiendesigns, die ein breiteres Spektrum von Patienten einschließen, eine geringere Wahrscheinlichkeit haben, signifikante Ergebnisse zu erzielen, was entsprechend größere Stichproben erfordert und die Kosten der Studien erhöht. Dies wiederum könnte zu Verzögerungen bei der Entwicklung neuer Medikamente führen. Um diesen Effekten zu begegnen, könnten (pragmatische) (Plattform)Studien auf Basis von Patientenregistern den Aufwand bei der Rekrutierung und Nachverfolgung reduzieren und werden 17 bspw. in Skandinavien bereits regelhaft umgesetzt 18 .

A-priori-Studien zur Generalisierbarkeit auf der Grundlage von Registerdaten und Informationen zum Studiendesign könnten eine Möglichkeit für Prüfer sein, Studien vor Beginn anzupassen. Post-market surveillance Studien und Posteriori-Studien zur Generalisierbarkeit wie die vorliegende können von einer Datenverknüpfung profitieren, bei der primäre Registerdaten und sekundäre Daten (z. B. Daten von Krankenversicherungen) zusammengeführt werden, um die klinische Versorgungsroutine und die behandelte Bevölkerung noch besser zu beschreiben. Eine Integration von Ergebnissen randomisierter klinischer Studien mit primärer erhobenen Registerdaten und Sekundärdatenquellen könnte zu einer Verbesserung des Lebenszyklus von Arzneimitteln im Hinblick auf eine sichere und effiziente Arzneimitteltherapie führen.

## Zusätzliches Material

Zusätzliches Material Zusätzliches Material
