# Supplementary material for: Generalisability of Phase III Clinical Trials Using the Example of Two German Multiple Sclerosis Registries
Source: Gesundheitswesen. 2025 Jul 23;87(12):767–76. [Article in German] doi: 10.1055/a-2540-1749 (PMC12674891; doi:10.1055/a-2540-1749)
Supplement: Supplementary file 1 — Zusätzliches Material [file 10-1055-a-2540-1749-gesu-2024-07-2091-oa.pdf]

Onlinematerial 1. Einschlusskriterien für klinische Studien der Phase III, die Informationen über die klinische Wirksamkeit und Sicherheit von krankheitsmodifizierenden Therapien für Patienten mit Multipler Sklerose liefern.

DMD	Alter	EDSS Score	Verlaufsform	Schübe
Ocrelizumab	18 - 55	0.0 - 5.0	RRMS	Mindestens 2 Schübe innerhalb der letzten 2 Jahre vor dem Randomisierung oder ein Schübe im Jahr vor dem Screening. Kein Schübe 30 Tage vor Studienbeginn.
Cladribin	18 - 65	0.0 - 5.5	RRMS	Mindestens ein Schübe innerhalb von 12 Monaten vor Studienbeginn. Kein Schübe innerhalb von 28 Tagen vor Studienbeginn.
Daclizumab	18 - 55	0.0 - 5.0	RRMS	Mindestens ein Schübe in den 12 Monaten vor der Randomisierung.
Dimetylfumarate	18 - 55	0.0 - 5.0	RRMS	Mindestens ein Schub innerhalb der letzten 12 Monate vor der Randomisierung.
Teriflunomid	18 - 55	0.0 - 5.5	RRMS/SPMS	Mindestens ein Schub in den letzten 12 Monaten oder mindestens 2 Schübe in den letzten 24 Monaten vor der Randomisierung. Kein Schub innerhalb von 30 Tagen vor der Randomisierung.
Alemtuzumab	18 - 55	0.0 - 5.5	RRMS	Mindestens zwei Schübe 2 Jahre vor Randomisierung und mindestens ein Schub im ein Jahr vor Randomisierung.
Fingolimod	18 - 55	0.0 - 5.5	RRMS	Mindestens zwei Schübe 2 Jahre vor Randomisierung und mindestens ein Schub im ein Jahr vor Randomisierung.
Natalizumab	18 - 50	0.0 - 5.0	RRMS	Mindestens ein Schub innerhalb der letzten 12 Monate vor Beginn der Studie. Kein Schub innerhalb von 50 Tagen vor der ersten Verabreichung des Studienmedikaments.
Mitoxantron	18 - 55	3.0 - 6.0	RRMS/SPMS	Keine Schübe 8 Wochen vor der Randomisierung.
Glatirameracetat	18 - 50	0.0 - 5.0	RRMS	Mindestens zwei Schübe in den letzten 2 Jahren vor der Aufnahme in die Studie, Beginn des ersten Schubs mindestens 1 Jahr vor der Randomisierung.

Das Gesundheitswesen | DOI 10.1055/a-2540-1749 | © The Author(s).

Peginterferon β-1a	18 - 65	0.0 -5.0	RRMS	Mindestens 2 Schübe 3 Jahre vor Studienbeginn, wobei mindestens ein Schübe in den letzten 12 Monaten aufgetreten sein muss.
Interferon β-1a	18 - 55	0.0 - 5.0	RRMS	Mindestens zwei Schübe 2 Jahre vor Randomisierung.
Interferon β-1b	18 - 55	0.0 - 5.5	RRMS	Mindestens zwei Schübe 2 Jahre vor Randomisierung.

DMD: disease-modifying drug; EDSS Score: expanded disability status scale score; RRMS: schubförmige remittierende MS; SPMS: sekundär progrediente MS;

Das Gesundheitswesen | DOI 10.1055/a-2540-1749 | © The Author(s).

Onlinematerial 2. Kriterien für die Aufnahme in eine klinische Studie der Phase III, die Informationen über die klinische Wirksamkeit und Sicherheit krankheitsmodifizierender Therapien für Patienten mit Multipler Sklerose liefert.

DMD	Medikationsgeschichte
Ocrelizumab	Keine Behandlung mit β-Interferonen, Glatirameracetat oder anderen immunmodulatorischen Therapien innerhalb von 4 Wochen vor Studienbeginn. Keine vorherige Behandlung mit zielgerichteten B-Zell-Therapien (Rituximab, Ocrelizumab) oder mit Alemtuzumab, Cladribin, Mitoxantron, Daclizumab und Teriflunomid. Keine Behandlung mit Cyclophosphamid, Azathioprin, Methotrexat oder Natalizumab innerhalb von 24 Monaten vor dem Screening. Patienten, die zuvor mit Natalizumab behandelt wurden, sind nur dann für die Studie zugelassen, wenn die Behandlungsdauer < 1 Jahr betrug. Keine Behandlung mit Fingolimod innerhalb von 24 Wochen vor Randomisiereung.
Cladribin	Bei Patienten, die ein DMD erhalten hatten, war eine mindestens dreimonatige Auszeit vor Studienbeginn erforderlich. Keine vorherige immunsuppressive Behandlung. Keine vorherige Behandlung mit Natalizumab.
Daclizumab	Keine vorherige Behandlung mit Caldribin. Keine vorherige Behandlung mit Mitoxantron, Cyclophosphamid, Fingolimod oder Natalizumab innerhalb eines Jahres vor der Randomisierung. Keine Behandlung mit Glatirameracetat innerhalb der letzten 30 Tage vor der Randomisierung.
Dimethylfumarat	Keine vorherige Behandlung mit Glatirameracetat oder Cladribin. Keine vorherige Behandlung mit Mitoxantron innerhalb eines Jahres vor der Randomisierung. Keine vorherige Behandlung mit Natalizumab innerhalb der letzten 6 Monate vor der Randomisierung. Keine vorherige Behandlung mit Interferonen innerhalb der letzten 3 Monate vor der Randomisierung. Keine Behandlung mit Steroiden oder oralen Kortikosteroiden innerhalb von 50 Tagen vor der Randomisierung.
Teriflunomid	Keine Therapie mit Glatirameracetat oder Interferonen 3 Monate vor der Randomisierung. Keine vorherige Anwendung von Natalizumab, Cladribin oder Mitoxantron.
Alemtuzumab	Keine Behandlung innerhalb der letzten 6 Monate vor Studienbeginn mit Natalizumab oder Methotrexat.
Fingolimod	Keine Anforderungen.
Natalizumab	Keine Behandlung mit Mitoxantron oder Cyclophosphamid innerhalb des letzten Jahres vor Randomisierung. Keine Behandlung mit Interferonen, Azathioprin, Glatirameracetat oder Immunglobulinen innerhalb der letzten 6 Monate vor Randomisierung.
Mitoxantron	Keine Behandlung mit Glukokortikosteroiden 8 Wochen vor Studeinbeginn. Keine vorherige Therapie mit Interferonen, Glatirameracetat oder Mitoxantron.

Das Gesundheitswesen | DOI 10.1055/a-2540-1749 | © The Author(s).

Glatirameracetat	Keine Anforderungen.
Peginterferon β-1a	Die Probanden müssen die Interferon-Behandlung 6 Monate vor Studienbeginn abgesetzt haben. Keine vorherige Behandlung mit Caldribin, Fingolimod oder Mitoxantron (1 Jahr vor Studienbeginn) und Glatirameracetat (4 Wochen vor Studienbeginn).
Interferon β-1a	Keine vorherige Behandlung mit Interferon oder mit anderen immunmodulatorischen oder immunsuppressiven Behandlungen12 Monate vor Randomisierung.
Interferon β-1b	Keine Anforderungen.

Das Gesundheitswesen | DOI 10.1055/a-2540-1749 | © The Author(s).
